# Anatomical and clinical factors associated with infrapopliteal arterial bypass outcomes in patients with chronic limb-threatening ischemia

**DOI:** 10.1007/s00380-024-02421-6

**Published:** 2024-06-06

**Authors:** Makoto Haga, Shunya Shindo, Jun Nitta, Mitsuhiro Kimura, Shinya Motohashi, Hidenori Inoue, Junetsu Akasaka

**Affiliations:** 1 Department of Cardiovascular Surgery, Tokyo Medical University Hachioji Medical Center, 1163 Tatemachi-chou, Hachioji-shi, Tokyo, 193-0944 Japan; 2 Center for Preventive Medicine, Yamanashi Kosei Hospital, Yamanashi, Japan

**Keywords:** Infrapopliteal arterial bypass, Limb-based patency, Global limb anatomic staging system, Severe calcification

## Abstract

The aim of this study was to identify anatomical and clinical factors associated with limb-based patency (LBP) loss, major adverse limb events (MALEs), and poor amputation-free survival (AFS) after an infrapopliteal arterial bypass (IAB) surgery according to the Global Limb Anatomic Staging System. A retrospective analysis of patients undergoing IAB surgery between January 2010 and December 2021 at a single institution was performed. Two-year AFS, freedom from LBP loss, and freedom from MALEs were assessed using the Kaplan–Meier method. Anatomical and clinical predictors were assessed using multivariate analysis. The total number of risk factors was used to calculate risk scores for subsequent categorization into low-, moderate-, and high-risk groups. IABs were performed on 103 patients. The rates of two-year freedom from LBP loss, freedom from MALEs, and AFS were 71.3%, 76.1%, and 77.0%, respectively. The multivariate analysis showed that poor run-off beyond the ankle and a bypass vein caliber of < 3 mm were significantly associated with LBP loss and MALEs. Moreover, end-stage renal disease, non-ambulatory status, and a body mass index of < 18.5 were significantly associated with poor AFS. The rates of freedom from LBP loss and MALEs and the AFS rate were significantly lower in the high-risk group than in the other two groups (12-month low-risk rates: 92.2%, 94.8%, and 94.4%, respectively; 12-month moderate-risk rates: 58.6%, 84.6%, and 78.3%, respectively; 12-month high-risk rates: 11.1%, 17.6%, and 56.2%, respectively; *p* < 0.001, *p* < 0.001, and *p* < 0.001, respectively). IAB is associated with poor clinical outcomes in terms of LBP, MALEs, and AFS in high-risk patients. Risk stratification based on these predictors is useful for long-term prognosis.

## Introduction

The growing interest in infrapopliteal (IP) revascularization has led to the development of the Global Limb Anatomic Staging System (GLASS). This scoring system marks a shift in the treatment of chronic limb-threatening ischemia (CLTI) from a lesion-based to a limb-based approach [1]. Recent studies have reported interesting findings regarding the associations of GLASS with bypass surgery and endovascular revascularization (ER) [2–11]. However, despite the wide acceptance of GLASS, the prognosis of patients undergoing infrapopliteal arterial bypass (IAB) surgery has not been adequately studied based on anatomic and clinical factors. Therefore, the aim of this study was to identify the factors associated with post-IAB limb-based patency (LBP) loss, major adverse limb events (MALEs), and poor amputation-free survival (AFS) to help vascular surgeons predict treatment outcomes based on preoperative data, select the most beneficial treatment, and perhaps better counsel patients on outcome expectations.

## Methods

### Patient selection and data collection

A retrospective study of patients undergoing initial IAB surgery at Tokyo Medical University Hachioji Medical Center from January 2010 to December 2021 was conducted. Patients with no angiographic images of the foot, no follow-up data, previous infrainguinal ER or functioning bypass grafts, or an unsalvageable foot (Wound, Ischemia, and foot Infection (WIfI) classification system stage 5) were excluded from the analysis. The study was approved by the Institutional Review Board of Tokyo Medical University (No. T2019-0201). Informed consent was waived because of the study’s retrospective design.

The analyzed data included baseline demographic characteristics, comorbidities, and procedural details. Follow-up data were collected through a review of the patients’ medical histories and records.

Ambulatory status was assessed preoperatively. Patients admitted on foot or with cane support were considered ambulatory, whereas patients using wheelchairs and bed-bound patients were considered non-ambulatory.

Controlling Nutritional Status (CONUT) scores were calculated to evaluate nutritional status [12]. Malnutrition was defined as a CONUT score of ≥ 9. Table 1 presents the methodology used to calculate the CONUT scores. A body mass index (BMI) of < 18.5 was considered to indicate underweight [13]. Malnutrition and underweight were included as variables in the risk factor analysis.

**Table 1:** Definition of CONUT score

Parameters	Normal	Light	Moderate	Severe
Serum albumin (g/dl)	≥ 3.5	3.0–3.49	2.5–2.9	< 2.5
Score	0	2	4	6
Total lymphocyte (count/mm^3^)	≥ 1600	1200–1599	800–1199	< 800
Score	0	1	2	3
Total cholesterol (mg/dl)	≥ 180	140–180	100–139	< 100
Score	0	1	2	3
CONUT score (total)	0–1	2–4	5–8	9–12
Assessment	Low	Intermediate	High	High

*CONUT* Controlling Nutritional Status

The calibers of the saphenous veins were measured preoperatively and intraoperatively using duplex ultrasound with the patients in the supine position to determine whether they exceeded 3 mm. The intraoperative measurements were performed after the saphenous vein was harvested.

### Limb classifications

Wound, perfusion, and infection data, along with catheter angiography performed at the time of revascularization, were used to assign WIfI and GLASS stages [1, 14]. Two experienced vascular and cardiovascular surgeons blinded to the treatment methods and clinical outcomes evaluated the angiographic images and graded the femoropopliteal (FP) and IP segments and the inframalleolar (IM)/pedal descriptor. Disagreements were resolved with the help of a third observer, and the final staging was established based on consensus. The GLASS staging inter- and intra-observer variability has been evaluated previously [3]. The target artery pathway (TAP) for the treated limb was identified through a review of the operative report. Following the Global Vascular Guidelines (GVG), severe calcification within the FP or IP segments of the TAP was defined as > 50% of the circumference and diffuse, bulky, or coral reef plaques likely to compromise the revascularization outcome [1].

### Surgical procedures and postoperative care

IAB was defined as any bypass performed with a distal anastomosis beyond the popliteal artery. All procedures were performed by operators who had performed more than 100 open bypasses. IAB surgery was selected as the initial revascularization procedure based on a surgeon’s inspection. ER was selected if no autologous vein was available. Concomitant aorto-iliac and FP diseased lesions were treated prior to IAB surgery. A saphenous vein was harvested if its diameter was > 2 mm. If the great saphenous vein was unusable, the small saphenous vein was selected. All bypasses were performed in reverse fashion. The bypass target sites were determined based on a relatively disease-free arterial segment with adequate run-off detected using preoperative computed tomography (CT) and catheter angiography. Graft flow was measured using a flowmeter immediately after anastomosis [15]. Patients undergoing IAB surgery received single antiplatelet therapy. However, patients with narrow vein conduits (< 3 mm) were treated with dual antiplatelet therapy or anticoagulant therapy per the surgeon’s preference. Duplex ultrasound and ankle–brachial pressure index measurements were performed one, three, and six months postoperatively and every six months thereafter. Patients with signs of CLTI or wound regression and asymptomatic patients with duplex ultrasound surveillance findings of stenosis (increased peak systolic velocity of > 300 cm/s) underwent CT angiography. If stenosis or graft occlusion was confirmed, ER was performed to salvage the vein graft. If ER failed, an IAB surgery was undertaken. Graft patency was defined as LBP. Previous studies have reported that the commonly used end points of primary, primary-assisted, and secondary patency have failed to precisely evaluate the hemodynamic results of ER [16–18]. Therefore, LBP has been used as a novel end point intended to be one of the primary outcomes for GLASS in the GVG [1]. LBP is used to assess the anatomic durability of the TAP revascularization strategy and is particularly relevant to multilevel ER [2]. Following the GVG, LBP loss was defined as occlusion or > 70% stenosis within the TAP in an imaging study, a re-intervention affecting any portion of the TAP, or hemodynamic failure (> 50% stenosis in the TAP with recurrent/unresolved symptoms or a decrease of ≥ 0.15 in the ankle–brachial index) [1]. MALEs were defined as major amputation, a new bypass, open bypass revision, or repeat angioplasty. Cardiac function was assessed based on the left ventricular ejection fraction (EF), determined using echocardiography. An EF of < 50% was considered to indicate heart failure [19].

### Statistical analysis

All statistical analyses were performed using IBM SPSS Statistics version 29.0 (IBM Corporation, Armonk, NY, USA). Baseline characteristics were compared using Student’s *t* test for continuous variables and Fisher’s exact test for categorical variables. The long-term outcomes were assessed using the Kaplan–Meier estimator and compared with the results of a log-rank test for LBP loss, MALEs, and AFS. Variables with* p* values of < 0.05 in univariate analysis were considered potential risk factors for LBP loss, MALEs, and poor AFS and included in multivariate Cox proportional hazards regression. The regression results were expressed as hazard ratios (HR) with 95% confidence intervals. In all statistical tests, *p* values of < 0.05 were considered statistically significant.

For each patient with CLTI, the first limb undergoing LBP was used in the analysis. In cases in which both limbs of a patient with CLTI were re-vascularized on the same day, the limb that was in poorer condition, as determined according to the Rutherford classification or hemodynamic criteria, was selected. These patients were evaluated in both univariate and multivariate analyses, as well as Kaplan–Meier analysis.

Risk stratification analysis was performed using scores based on the number of variables independently associated with LBP loss, MALEs, and poor AFS in the multivariate analysis. The total number of risk factors was used to calculate risk scores and categorize the patients into low-risk (score of 0), moderate-risk (score of 1), and high-risk groups (score of 2 or 3). The long-term outcomes of risk categorization were assessed using the Kaplan–Meier estimator and compared with the results of a log-rank test for LBP loss, MALEs, and AFS.

## Results

A total of 103 patients undergoing IAB surgery were included in the analysis. The mean follow-up time was 48.1 ± 41.9 months. The patients’ demographic and clinical characteristics are summarized in Table 2. The average patient age was 71.2 ± 8.8 years. Most patients were male (71.8%). The malnutrition rate was 9.7%. Of the 103 patients, 15.5% were non-ambulatory. Of the 103 limbs, 22 (21.4%) had rest pain, 69 (67.0%) exhibited minor tissue loss, and 12 (11.7%) exhibited major tissue loss. GLASS stage III (85.4%) and a P1 IM/pedal descriptor (64.1%) were frequent. ER of more proximal lesions prior to IAB surgery was performed on 29.1% of cases, involving concomitant aorto-iliac (7.8%) and FP (21.3%) diseased lesions. More than half of the patients (57.3%) showed severe calcification. The two main inflow arteries were the femoral (48.5%) and popliteal arteries (51.5%). Detailed information on the outflow arteries is provided in Table 3. In 92.2% of the cases, a single-segment great saphenous vein was used as a conduit. The rate of vein calibers under 3 mm was 19.4%.

**Table 2:** Baseline characteristics for the infrapopliteal arterial bypass

Variable	Patients *n* = 103 (%)
Age, years	71.2 ± 8.8
Male gender	74 (71.8)
Body mass index	22.4 ± 3.9
Preprocedural ABI	0.53 ± 0.19
Postprocedural ABI	0.79 ± 0.18
Ex or current smoker	47 (45.6)
Diabetes mellitus	76 (73.8)
Hypertension	90 (87.4)
Hyperlipidemia	50 (48.5)
Ejection fraction	60.6 ± 10.5
Coronary artery disease	29 (28.2)
COPD	12 (11.7)
Cerebrovascular disease	13 (12.6)
Chronic renal insufficiency	83 (80.6)
End-stage renal disease	56 (54.4)
Nutritional status	
Malnutrition	10 (9.7)
Functional status	
Non-ambulatory	16 (15.5)
Preprocedural medication	
Antiplatelet	93 (90.3)
Statin	43 (41.7)
β-blocker	48 (46.6)

*ABI* ankle-brachial index, *COPD* chronic obstructive pulmonary disease

Chronic renal insufficiency is defined as an estimated glomerular filtration rate of < 50 mL/min/1.73 m^2^

Categorical variables are presented as numbers (percentages)

Continuous variables are presented as means ± standard deviations

**Table 3:** Distribution of Wound, Ischemia, and foot Infection (WIfI) and Global Anatomic Staging System (GLASS) scores and treatment details for the infrapopliteal arterial bypass

Variable	Overall, *n* = 103 (%)
Rutherford classification	
4 (Rest pain)	22 (21.4)
5 (Minor tissue loss—non-healing ulcer, focal gangrene with diffuse pedal ischemia)	69 (67.0)
6 (Major tissue loss—extending above TM level, functional foot no longer salvageable)	12 (11.7)
WIfI classification	
Wound (W)	
W0	18 (17.5)
W1	50 (48.5)
W2	25 (24.3)
W3	10 (9.7)
Ischemia (I)	
I0	1 (1.0)
I1	30 (29.1)
I2	38 (36.9)
I3	34 (33.0)
Foot infection (fI)	
fI0	20 (19.4)
fI1	52 (50.5)
fI2	25 (24.3)
fI3	6 (5.8)
WIfI stage	
1	0
2	28 (27.2)
3	39 (37.9)
4	36 (35.0)
GLASS	
FP grade	
0	21 (20.4)
1	15 (14.6)
2	11 (10.7)
3	20 (19.4)
4	36 (35.0)
IP grade	
0	1 (1.0)
1	25 (24.3)
2	11 (10.7)
3	23 (22.3)
4	43 (41.7)
GLASS Stage	
I	0 (0)
II	15 (14.6)
III	88 (85.4)
IM/pedal descriptor	
P0	18 (17.5)
P1	66 (64.1)
P2	19 (18.4)
Proximal intervention undertaken	30 (29.1)
Iliac lesion	8 (7.8)
FP lesion	22 (21.3)
Severe calcification	59 (57.3)
Graft flow (mL/min)	42.5 ± 34.7
Inflow artery	
Femoral artery	50 (48.5)
Popliteal artery	53 (51.5)
Outflow artery	
Below knee popliteal	23 (22.3)
Anterior tibial artery	11 (10.7)
Posterior tibial artery	25 (24.3)
Peroneal artery	7 (6.8)
Dorsalis pedis artery	27 (26.2)
Plantar artery	10 (9.7)
Type of bypass graft	
Single vein	95 (92.2)
Spliced vein	8 (7.8)
Conduit	
Great saphenous vein	102 (99.0)
Small saphenous vein	1 (1.0)
Vein caliber	
< 3.0 mm	20 (19.4)

All values are numbers (percentages)

*FP* femoropopliteal, *GLASS* global anatomic staging system, *IM* inframalleolar; *IP* infrapopliteal, *TM* transmetatarsal, *WIfI* wound, ischemia, and foot infection

The rates of freedom from LBP loss were 75.5% at one year, 71.3% at two years, and 70.2% at five years. The rates of freedom from MALEs were 79.3% at one year, 76.1% at two years, and 73.8% at five years. The overall AFS rates were 81.8% at one year, 77.0% at two years, and 66.0% at five years.

A univariate analysis showed that non-ambulatory status (*p* < 0.001), the absence of a P0 or P1 IM/pedal descriptor (*p* < 0.001), and a bypass vein caliber of less than 3 mm (*p* < 0.001) were associated with LBP loss. GLASS stage, WIfI classification stage 4, and severe calcification were not independently associated with LBP loss. In multivariate analysis, the absence of a P0 or P1 IM/pedal descriptor (HR 17.27 (4.09–72.89), *p* < 0.001) and a bypass vein caliber of less than 3 mm (HR 5.56 (1.81–17.01), *p* < 0.001) were independent predictors of LBP loss (Table 4). A univariate analysis showed that non-ambulatory status (*p* < 0.001), the absence of a P0 or P1 IM/pedal descriptor (*p* < 0.001), and a vein caliber of less than 3 mm (*p* < 0.001) were associated with MALEs. In multivariate analysis, non-ambulatory status (HR 4.84 (1.26–18.49), *p* = 0.021), the absence of a P0 or P1 IM/pedal descriptor (HR 12.16 (3.29–44.97), *p* < 0.001), and a vein caliber of less than 3 mm (HR 3.56 (1.19–10.63), *p* = 0.023) were independent predictors of MALEs (Table 4). A univariate analysis showed that a BMI of < 18.5 (*p* = 0.02), previous or current smoking status (*p* = 0.036), hypertension (*p* = 0.014), chronic obstructive pulmonary disease (*p* = 0.023), end-stage renal disease (ESRD) (*p* = 0.01), non-ambulatory status (*p* < 0.001), the absence of a P0 or P1 IM/pedal descriptor (*p* = 0.03), and severe calcification (*p* = 0.006) were associated with poor AFS. In multivariate analysis, a BMI of < 18.5 (HR 4.56 (1.38–15.01), *p* = 0.012), ESRD (HR 3.35 (1.35–8.36), *p* = 0.009), and non-ambulatory status (HR 6.64 (1.65–26.70), *p* = 0.008) were independent predictors of poor AFS (Table 4).

**Table 4:** Univariable and multivariate analysis of risk factors influencing LBP, MALEs, and AFS

Variables	LBP			MALEs			AFS		
	Univariable	Multivariate	HR (95% CI)	Univariable	Multivariate	HR (95% CI)	Univariable	Multivariate	HR (95% CI)
Male gender	0.42			0.32			0.38		
Age > 80	0.36			0.23			0.38		
Body max index < 18.5	0.36			0.28			0.02	0.012	4.56 (1.38–15.01)
Ex or current smoker	0.15			0.15			0.036	0.26	
Hypertension	0.42			0.25			0.014	0.16	
Hyperlipidemia	0.41			0.26			0.23		
Diabetes mellitus	0.30			0.38			0.21		
EF < 50	0.12			0.072			0.25		
Coronary artery disease	0.42			0.17			0.072		
Cerebrovascular disease	0.07			0.069			0.129		
COPD	0.49			0.42			0.023	0.088	
Chronic renal insufficiency	0.10			0.45			0.19		
End-stage renal disease	0.42			0.24			0.01	0.009	3.35 (1.35–8.36)
Malnutrition	0.31			0.26			0.13		
Non-ambulatory	< 0.001	0.053		< 0.001	0.021	4.84 (1.26–18.49)	< 0.001	0.008	6.64 (1.65–26.70)
WIfI stage 4	0.087			0.10			0.17		
GLASS stage 2 or 3	0.28			0.34			0.19		
IM/pedal descriptor	< 0.001	< 0.001	17.27 (4.09–72.89)	< 0.001	< 0.001	12.16 (3.29–44.97)	0.03	0.30	
Severe calcification	0.41			0.23			0.006	0.32	
Vein caliber < 3.0 mm	< 0.001	0.003	5.56 (1.81–17.01)	< 0.001	0.023	3.56 (1.19–10.63)	0.45		

All values are numbers (percentages)

*AFS* amputation-free survival, *COPD* chronic obstructive pulmonary disease, *EF* ejection fraction, *GLASS* global anatomic staging system, *IM* inframalleolar, *LBP* limb-based patency, *MALEs* major adverse limb events, *WIfI* wound, ischemia, and foot infection

**Fig. 1:**
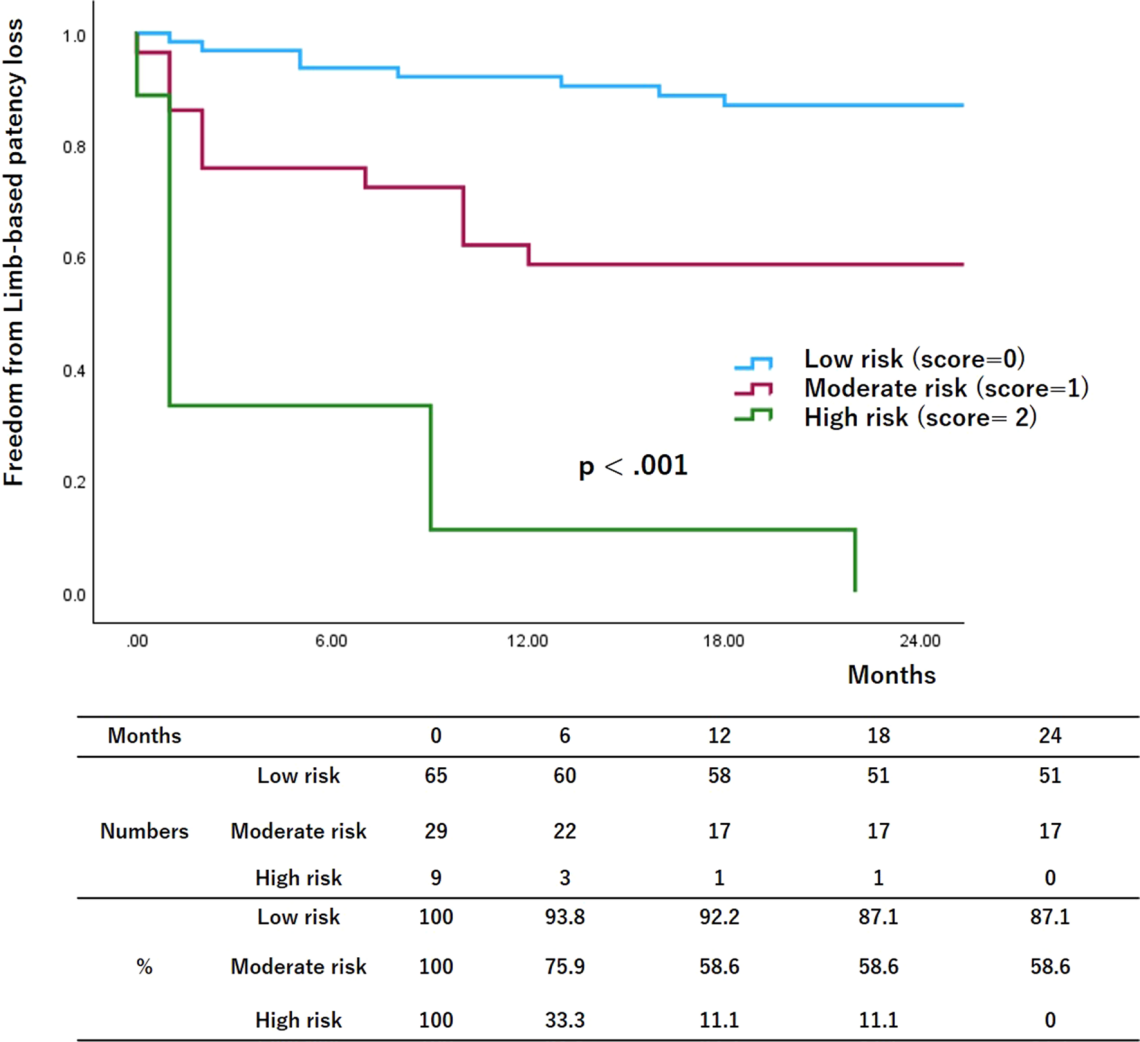
Risk stratification of limb-based patency (LBP) based on multivariate logistic regression analysis. The patients were assigned to groups based on the number of risk factors. The rates of freedom from LBP loss were lower in higher-risk groups

**Fig. 2:**
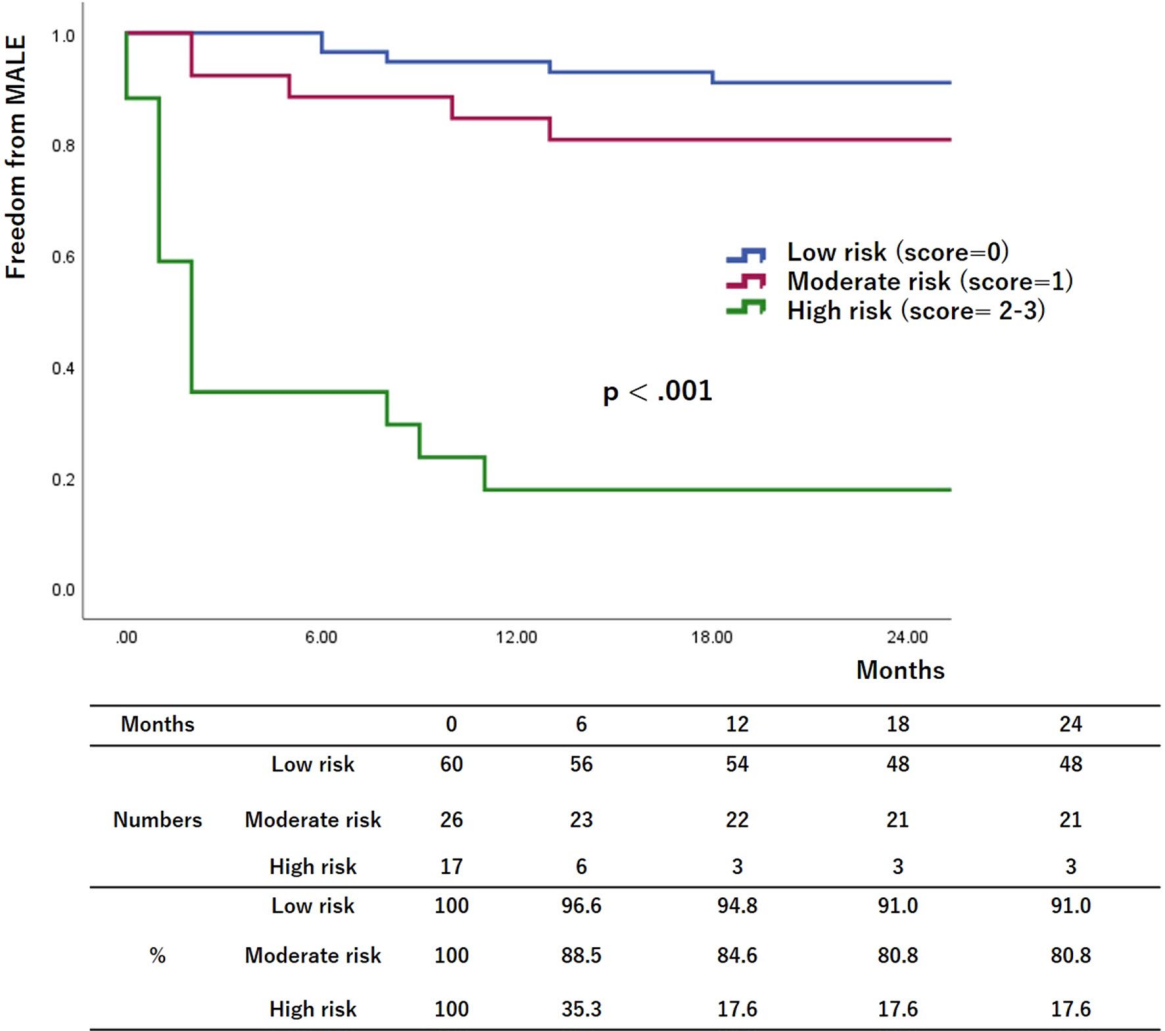
Risk stratification of major adverse limb events (MALEs) based on multivariate logistic regression analysis. The patients were assigned to groups based on the number of risk factors. The rates of freedom from MALEs were lower in higher-risk groups

**Fig. 3:**
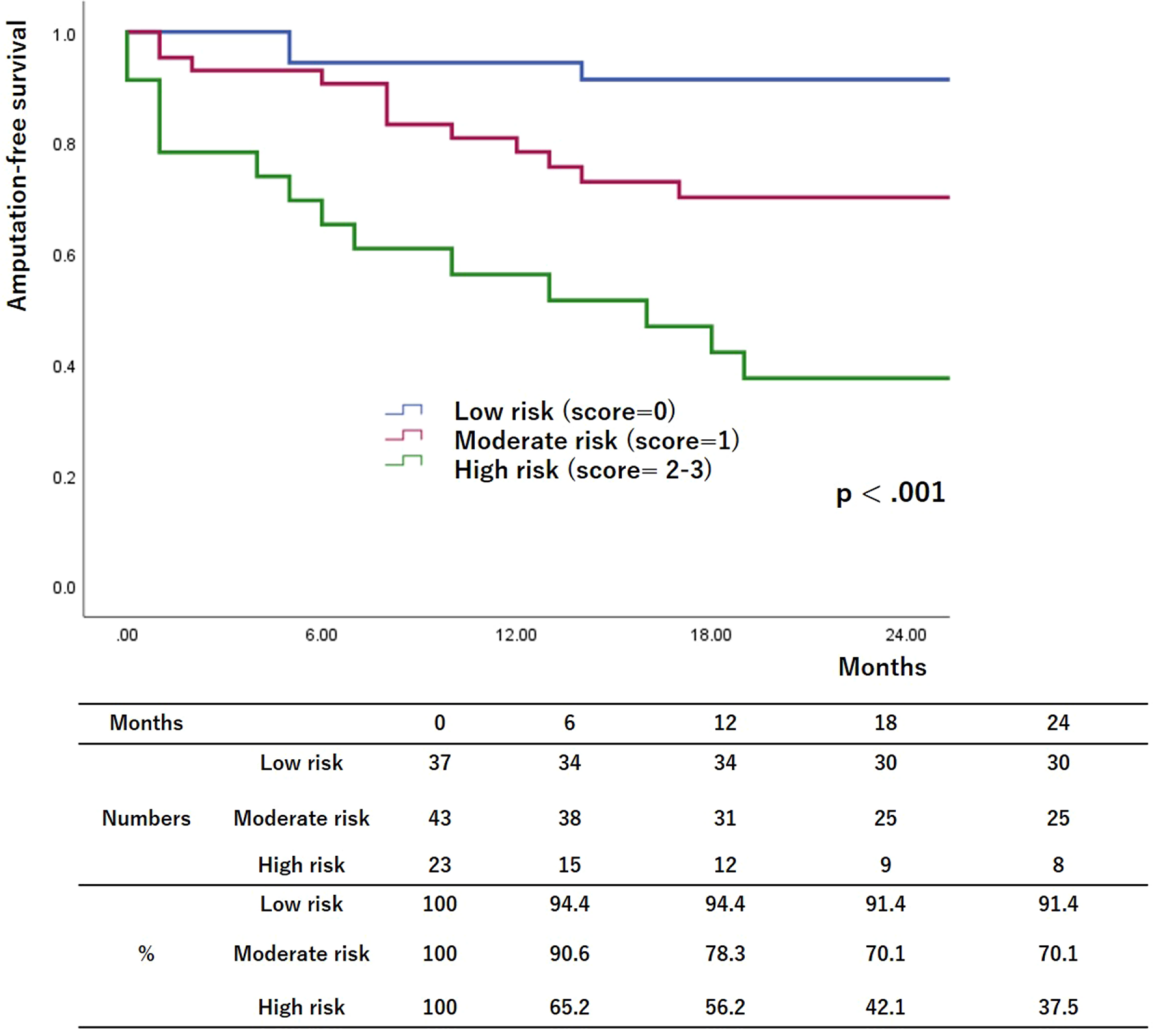
Risk stratification of amputation-free survival (AFS) based on multivariate logistic regression analysis. The patients were assigned to groups based on the number of risk factors. The AFS rates were lower in higher-risk groups

Figures 1, 2, and 3 show the risk stratifications of LBP loss, MALEs, and AFS according to the number of risk factors after the multivariate logistic analysis. The rates of freedom from LBP loss and MALEs and the AFS rate were significantly lower in the high-risk group than in the other two groups and in the moderate-risk group than in the low-risk group (12-month low-risk rates: 92.2%, 94.8%, and 94.4%, respectively; 12-month moderate-risk rates: 58.6%, 84.6%, and 78.3%, respectively; 12-month high-risk rates: 11.1%, 17.6%, and 56.2%, respectively; *p* < 0.001, *p* < 0.001, and *p* < 0.001, respectively).

## Discussion

In this single-center retrospective study, poor run-off beyond the ankle and a vein graft of less than 3 mm were significant independent predictors of LBP loss and MALEs after IAB surgery. Moreover, poor ambulatory status, ESRD, and a BMI of < 18.5 were significant independent predictors of poor AFS. A risk stratification analysis based on risk scores according to the number of predictive end points showed that the rates of LBP loss, MALEs, and poor AFS were higher in the high-risk group than in the moderate- and low-risk groups.

GLASS is an anatomical staging system designed as a clinical decision-making tool for selecting either bypass surgery or ER. Although many studies have reported its efficacy [2–11], few studies have assessed its association with IAB outcomes. The associations of GLASS grades with LBP loss, MALEs, and poor AFS observed in this study suggest that the main anatomical complexity does not correlate with these factors after IAB surgery. Moreover, inadequate run-off beyond the ankle (P2 descriptor) appears to be the only GLASS factor associated with LBP loss and MALEs. As several studies on IM/pedal descriptors have shown, evaluating run-off beyond the ankle is challenging. Hata et al. reported that poor run-off below the ankle was significantly associated with delayed wound healing after ER [4]. Liang et al. reported that the IM/pedal descriptor was the only GVG descriptor significantly associated with major amputation [7]. Kobayashi et al. found that a P2 descriptor was associated with lower primary patency and wound healing after IAB surgery [20]. In line with these findings, our results suggest that the IM/pedal descriptor is useful for assessing the flow profile preoperatively. In the current GVG, this descriptor does not alter the GLASS stage or the recommendation for revascularization. Thus, we believe that the IM grade is an important addition to revascularization decision-making and should be considered along with GLASS, especially when an IAB surgery is performed.

Whether tibial arterial calcification contributes to poor patency has been the subject of debate. However, it is clear that arterial calcification is associated with an increased risk of adverse limb events, including amputation [21, 22], and worsening limb ischemia [23]. The problem is that some surgeons consider such patients ineligible for revascularization, while others believe that a lower extremity bypass is possible [24, 25]. More than half of our patients showed severe calcification, which is typical of Japanese CLTI patients [3, 26]. However, we did not find severe calcification to be a predictor of LBP loss or MALEs. In contrast, in the endovascular field, lesion calcification is considered a predictor of MALEs in patients undergoing IP ER [27, 28]. Moreover, the degree of IP calcification is a significant predictor of wound healing after IP ER [4]. Severe calcification influences LBP more strongly after IP ER than after an IAB surgery. This may be because ESRD exacerbates atherosclerosis in the IP region [29]; thus, during ER, lesion calcification encourages overly aggressive balloon dilatation, leading to restenosis and compromised blood flow to the ischemic wound [28]. This occurs particularly often in IP legions, and there is general agreement that the rate of restenosis after ER in IP legions is extremely high (73% at three months) [30]. One can argue for the superiority of IAB surgery when the run-off below the ankle is adequate and when the anastomosis site is not severely calcified. If the anastomosis site is severely calcified, using the fracture technique to overcome the rigidity of the arterial wall [31, 32], selecting a less calcified lesion for anastomosis, and performing local endarterectomy (if all other options fail) may supplementarily maintain LBP for an IAB surgery, which is a common strategy in current vascular surgical practice.

In this study, all patients had CLTI. CLTI attributable to IP lesions is considered the most severe form of peripheral artery disease, and treatment management is challenging, especially for patients with impaired ambulatory capacity. Therefore, vascular surgeons always face a dilemma about whether to perform revascularization on any non-ambulatory patient. Prior studies have reported that ambulatory status is a significant predictor of mortality after bypass surgery [33–36]. Even when a minimally invasive option, such as ER, is selected for non-ambulatory patients, the perioperative mortality and adverse event rates are still high [37]. In line with this important finding, our study shows that non-ambulatory status is a strong predictor of MALEs and poor AFS after an IAB surgery, suggesting that vascular surgeons should be extremely cautious about performing revascularization on non-ambulatory patients.

In line with our findings, previous studies have shown that veins with a diameter of < 3 mm are associated with worse patency [38–40]. Interestingly, however, we did not find an association between vein diameter and AFS. This suggests that when an IAB is successfully performed on a patient with CLTI, even if LBP declines or a re-intervention is needed, a small vein diameter (2–3 mm) is not associated with subsequent adverse events, including amputation and death. In this study, we assessed vein diameter but did not thoroughly evaluate vein quality. Preoperative duplex ultrasound may be useful for determining whether the saphenous vein is fragile or thick. If the quality of a vein is normal, it may be usable as a bypass graft even if its diameter is small. In the BEST-CLI study, among patients with adequate great saphenous veins, the rates of MALEs and death were lower in the bypass group than in the ER group [41]. As the importance of an adequate saphenous vein is increasingly recognized, establishing an accurate surveillance protocol or a scoring system for bypass vein grafts is necessary for reducing the incidence of LBP loss and MALEs.

CONUT has been reported to be the most effective nutritional scoring system for predicting survival and wound healing in patients with CLTI [42]. However, in this study, CONUT was not associated with limb or life prognosis. One reason might be the relatively small sample size. Another potential factor is that we did not use the Geriatric Nutritional Risk Index (GNRI), which is also a highly reliable nutritional scoring system for predicting survival in patients with CLTI [43]. Given that the GNRI incorporates BMI into its calculation, it may serve as an independent predictor of poor AFS. At any rate, since a low BMI showed an association with AFS, it can be used as a complementary measure for assessing limb or life prognosis.

Certain limitations of this study should be noted. This was a retrospective single-center study with a relatively small sample size. Furthermore, we did not evaluate postoperative wound healing or changes in the WIfI scores. Moreover, the degree of postoperative rehabilitation is subject to assessment bias. Another limitation is that ambulatory status can be a subjective measure and may change postoperatively due to CLTI. Despite these limitations, this study obtained important findings regarding the predictors of LBP loss, MALEs, and poor AFS after an IAB, which can help inform the selection of an IAB strategy. Further studies with larger and more heterogeneous cohorts are needed to clarify the risk factors for LBP loss, MALEs, and poor AFS.

## Conclusions

Poor run-off beyond the ankle and a bypass vein caliber of < 3 mm are associated with LBP loss and MALEs after an IAB. End-stage renal disease, non-ambulatory status, and a BMI of < 18.5 are associated with poor postoperative AFS. Risk stratification analysis based on these predictors can play an important role in estimating future LBP loss, MALEs, and poor AFS in patients with CLTI undergoing IAB surgery and can help vascular surgeons predict surgical outcomes and conduct better patient selection.

## Data Availability

The data that support the findings of this study are available from the corresponding author, upon reasonable request.

## Declarations

### Presentation information

This study was presented at the 35th Annual Meeting of the European Society for Vascular Surgery, Rotterdam, 20 September 2021.
